# Effect of lignin-blocking agent on enzyme hydrolysis of acid pretreated hemp waste

**DOI:** 10.1039/d1ra03412j

**Published:** 2021-06-22

**Authors:** Daehwan Kim, Chang Geun Yoo, Jurgen Schwarz, Sadanand Dhekney, Robert Kozak, Craig Laufer, Drew Ferrier, Skylar Mackay, Madyson Ashcraft, Richard Williams, Sinyeon Kim

**Affiliations:** Department of Biology, Hood College Frederick MD 21701 USA kimd@hood.edu; Department of Chemical Engineering, State University of New York – College of Environmental Science and Forestry Syracuse NY 13210 USA; Department of Agriculture, Food and Resource Sciences, University of Maryland Eastern Shore Princess Anne MD 21853 USA; Atlantic Biomass Conversions, LLC Frederick MD 21701 USA; MtheraPharma Co., Ltd. Seoul 07793 Republic of Korea

## Abstract

Hemp wastes (stems and branches), fractionated after hemp flower extraction for the production of cannabidiol oil, were utilized as a potentially renewable resource for the sugar flatform process. Hydrolysis of cellulose from the acid pretreated hemp biomass using a commercial enzyme was tested and evaluated for its chemical composition, morphological change, and sugar recovery. Acid pretreated hemp stems and branches, containing 1% glucan (w/v) solids, were hydrolyzed for 72 h using 25 mg enzyme protein per g glucan. A 54% glucose conversion was achieved from the treated branches *versus* a 71% yield from the treated stems. Raw branches and stems yielded 35% and 38% glucose, respectively. Further tests with a lignin-blocking additive (*e.g.* bovine serum albumin) resulted in a 72% glucose yield increase for stem hydrolysis using 10 mg enzyme protein per g glucan. While pretreatment promotes amorphous hemicellulose decrease and cellulose decomposition, it causes enzyme inhibition/deactivation due to potential inhibitors (phenols and lignin-derived compounds). This study confirms the addition of non-catalytic proteins enhances the cellulose conversion by avoiding non-productive binding of enzymes to the lignin and lignin-derived molecules, with lignin content determining the degree of inhibition and conversion efficiency.

## Abstract 2

Enzymatic hydrolysis of acid pretreated hemp wastes is evaluated for its chemical composition, structural change, and sugar recovery. Addition of BSA enhances the cellulose conversion by avoiding non-productive binding between enzymes and inhibitors.

## Graphical abstract

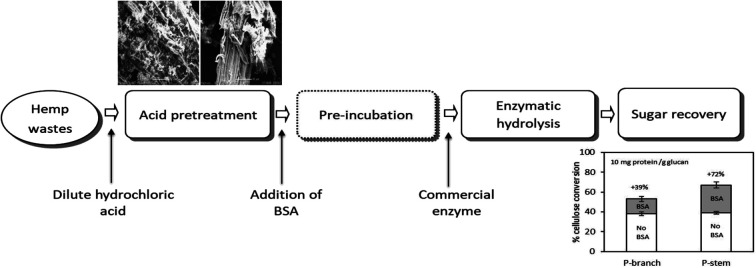

## Introduction

Hemp, a class of *Cannabis sativa* plant species, has been widely consumed as food, nutrition source, and pharmaceutical, and used in diverse industries such as paper, textile, plastic, construction, and wastewater purification.^1,2^ In particular, *cannabis* plants are currently receiving attention as a medicinal drug, which has positive effects on mental and physical conditions such as anxiety, euphoria, relaxation, damaged short-term memory, muscular movement, and behavior problems.^3^ While industrial hemp has been grown worldwide for various purposes, the private/commercial cultivation of industrial hemp has been subject to legal restrictions in most countries for several decades due to the drug abuse, misuse, and inebriating component of delta-9 tetrahydrocannabinol (THC) in marijuana.^4–6^

After the Federal Marijuana Prohibition Act on industrial hemp cultivation was lifted, the interest and demand for hemp and hemp-related markets for food, pharmaceutical, and biochemical products have rapidly increased. Industrial hemp is mostly used for the production of oils extracted from flowers (cannabidiol oil, cosmetics, pharmaceuticals), hempseed chemicals (food, meal, cosmetics), roots (phytoremediation, revitalization), and fiber applications; however, other components such as hurds, stems, branches, and leaves are not fully utilized. Most states (excluding Idaho, Mississippi, and the District of Colombia, https://www.ncsl.org) allow cultivation, harvest, and processing of industrial hemp (<0.3% THC per dry weight basis) under state agricultural hemp pilot research programs and the Department of Agriculture.^7^

Hemp biomass is primarily composed of cellulose, hemicellulose, and lignin with ash and extractives.^8^ High polysaccharide (30–50% of cellulose and 10–20% of hemicellulose) contents in hemp plants are comparable to those in other herbaceous or woody plants. A comparison of hemp with common lignocellulosic biomass types is summarized in Table 1A. Given hemp's chemical composition, broad industrial hemp cultivation, and the uncertain transfer of hemp products between states in the U.S., hemp wastes have been considered as alternative sustainable and renewable energy sources. Several studies indicated that hemp biomass has more cellulose components and less lignin, which is amenable to the production of fermentable sugars and transportation biofuel.^9–11^ However, some recalcitrance factors, such as the crystallinity of cellulose in hemp tissues, cause technical obstacles in utilization; therefore, pretreatment is essential to remove/reduce hemicellulose and/or lignin and increase cellulose accessibility to alleviate the biomass recalcitrance factors.^12–15^ Several studies have reported different pretreatment methods to improve sugar yields, including dilute acid,^10,16–19^ hydrothermal-mechanical,^20^ liquid hot water,^7^ steam explosion,^19,21^ deep eutectic solvents,^22,23^ and ionic liquid.^24^ The increase in sugar yield depends on the pretreatment condition and its subsequent process in cellulolytic saccharification. Das *et al.* (2020) found that both dilute acid (1% w/v H_2_SO_4_, 140 °C for 30 min) and alkali (2% w/v NaOH, 140 °C for 60 min) pretreatments were effective at obtaining a high glucose yield (>90%) from industrial hemp.^25^ Similarly, an attempt with acid-assisted steam pretreatment (210 °C with 2% SO_2_ impregnation) for industrial hemp resulted in a high glucose yield of 89%.^18,19^ More recent work done by Zhao *et al.* (2020) compared liquid hot water (LHW), H_2_SO_4_, and NaOH pretreatment and elucidated the advantages and disadvantages of each method. They observed that NaOH pretreatment was capable of increasing glucose yield up to 88.9%, which was significantly higher than those from acid (41.7–58.7%) and LHW (59.1–71.7%) pretreatment by achieving higher glucan composition and lignin removal.^7^ A high severity of NaOH pretreatment and its swelling effect in alkaline solution where keeps the external surface in the solids contribute the lignin removal and glucose conversion than those from other pretreatment methods. Even though alkali pretreatment showed a better conversion yield in the previous work, dilute acid treatment was selected for our current study to avoid the formation of lignin-derived products and to evaluate the effect of a bovine serum albumin supplement on insoluble lignin and lignin-derived molecules in the different pretreated hemp fractions of stem and branch.

(A) Chemical compositions of common plant biomass types, and (B) strategies to minimize deleterious inhibitors of enzymatic hydrolysis

**Table:** 

(A)	Composition (%)			
Biomass	Cellulose	Hemicellulose	Lignin	Ref.
Industrial hemp	32.6–44.5	16.6–15.5	17.0–21.5	20
Corn stover	37.0	22.7	18.6	12
Sugarcane bagasse	37.9	21.5	27.2	26
Hardwood	39.8	16.6	31.0	27
Corn pericarp	22.5	23.7	4.7	28
Switchgrass	39.5	10.3	17.8	29
Poplar	43.8	14.8	29.1	30

**Table:** 

(B) Strategy	Main effect	Ref.
Conditioning	Remove soluble inhibitors with chemical supplementations	27 and 31
Biological detoxification	Metabolize undesirable molecules using microbes	12, 13 and 32
Biomass selection and metabolic engineering	Screen adequate crops and/or genetically modify selected feedstock	33–36
Simultaneous saccharification and fermentation (SSF)	Minimize the product inhibition (glucose or other monomers)	37 and 38
Non-specific blocking agent	Increase enzyme accessibility by adding BSA, soybean protein or additives	26 and 39–41

Pretreatment of lignocellulosic materials primarily increases internal components of cellulose by solubilizing hemicellulose and lignin, as well as increases surface area and porosity that facilitate cellulose decomposition with cellulolytic enzymes.^42–44^ While pretreatment is an essential step for the use of lignocellulosic biomass, it prompts the generation of toxic molecules, which interrupt enzyme exposure to cellulose and microbial cell viability for the fermentation process.^45^ It is well-known that potential soluble inhibitors/deactivators include phenols, lignin-derived molecules, acetic acid, furan aldehydes, carboxylic acids, oligosaccharides, and degraded intermediates. Many approaches have been studied to identify inhibitors, minimize their formation during pretreatment, alleviate their negative effects on cellulase hydrolysis and/or microbial fermentation. For example, hot water washing was cost-effective to remove soluble inhibitors, and the addition of activated carbon and a blocking agent (*e.g.*, bovine serum albumin or soybean protein) could reduce enzyme inhibition by lignin or lignin-derived compounds. Other studies described various approaches such as liquid–liquid extraction,^46^ microbial detoxification,^12,13,47^ and genetic modification of low lignin crop^26^ to minimize the non-productive binding of enzymes and potential inhibitory effects. Detoxification methods are briefly summarized in Table 1B.

Changes in chemical composition and morphology in lignocellulosic biomass before/after pretreatment are used to determine the effect of selected pretreatment; however, the impacts of those pretreatments on hemp characteristics and conversion have not been fully elucidated. Therefore, this current work examined structural and compositional changes in hemp waste (stems and branches) before and after acid pretreatment. The changes in the complex structure of hemp samples during pretreatment were captured *via* scanning electron microscopy (SEM), and subsequent cellulolytic hydrolysis of cellulose in a hemp slurry was tested at different enzyme loadings. Furthermore, the addition of a lignin-blocking additive (bovine serum albumin, BSA) was applied to pretreated hemp solids to improve cellulose conversion to glucose, and its efficacy was compared with those without the BSA addition.

## Experimental

### Materials

Industrial hemp was cultivated at the Chicken of the Woods Permaculture Farm (Gaithersburg, MD, 39°07′02.4′′N latitude, 77°16′46.9′′W longitude, https://chickenwoods.com) in 2019, and they kindly donated hemp stems and branches after harvest and extraction of flowers for their market business. Hemp samples were dried in an oven overnight at 50 °C and ground using a Wiley mill with a 30-mesh screen (0.595 mm). The milled stem and hemp were then collected and oven-dried for further use. The moisture content of each sample was measured (<3%) using a Halogen moisture analyzer (Mettler Toledo HB 43, Columbia, OH, USA). Cellic CTec2 (enzyme blend, SAE0020) and Cellulase (Celluclast 1.5L, C2730) were purchased from Sigma-Aldrich (St. Louis, MO). Multipect pectinase enzyme was provided from Genencor, Danisco Division (Palo Alto, CA). All chemical reagents such as hydrochloric acid and 72% sulphuric acid (H_2_SO_4_) were purchased from Sigma-Aldrich (St. Louis, MO) unless otherwise indicated.

### Acid pretreatment

Hemp biomass at 15% (w/v) was pretreated in screw-capped glass bottles, as previously described in Ko *et al.* (2018).^48^ Approximately a 33 g (dry weight) hemp sample suspended in 220 mL of a 1% (v/v) hydrochloric acid solution was autoclaved at 121 °C for 30 min. The pretreated solids were vacuum filtered *via* a Buchner funnel onto a Whatman #1 filter paper (Whatman International Ltd., Springfield, England, Cat. no 1001125). Solid-free liquid obtained from the initial filtration was collected and filtered through a 0.45 μm nylon syringe filter (Acrodisc, Cortland, NY, USA) for further HPLC analysis of sugars and acids. The vacuum filtered solids on the filter paper were washed with 100 mL of distilled water (room temperature) to remove soluble inhibitors. The washing step was conducted in triplicate, and washed materials were oven-dried overnight. The dry weight of pretreated and washed stem and branch solids were 18.4 g and 22.6 g, corresponding to 55.8% and 68.6% dry solids/initial dry solids content, respectively. The changes of chemical compositions in hemp samples before and after pretreatment were determined as followed by the National Renewable Energy Laboratory (NREL) LAP standard analytical protocols.^49^ Comparisons between raw and pretreated hemp solids were presented in Table 2A. The derived phenolic acids formed during pretreatment were identified through a Folin-Ciocalteu colorimetry method with a spectrophotometer at 765 nm (Table 2B).

(A) Chemical composition of hemp stems and branches before and after pretreatment and (B) sugars and soluble inhibitors after pretreatment and vacuum filtered liquid fraction. For pretreatment, 15% (w/v) hemp biomass in the presence of 1% (v/v) hydrochloric acid was autoclaved at 121 °C for 30 min. All tests were completed in duplicate

**Table:** 

(A)	Composition (% by dry weight basis)			
	Stem		Branch	
	Raw	Pretreated	Raw	Pretreated
Glucan	39.6 ± 0.11	52.6 ± 1.08	26.0 ± 1.49	34.1 ± 0.34
Xylan	22.3 ± 0.28	10.6 ± 0.45	12.0 ± 1.64	11.5 ± 0.22
Arabinan	—	—	0.15 ± 0.22	—
Lignin	26.0 ± 0.21	29.4 ± 0.42	30.6 ± 0.58	35.6 ± 0.99
Solids recovery	55.8	—	68.6	—

a Not detected.

b Folin–Ciocalteu colorimetry assay at 765 nm.

**Table:** 

(B)	Pretreatment liquid of hemp (g L^−1^)	
	Stem	Branch
Glucose	0.66	8.46
Xylose	0.17	0.8
HMF	N.D.^a^	N.D.^a^
Furfural	N.D.^a^	N.D.^a^
Total phenols (mg L^−1^)^b^	323.8	387.5

### Enzymatic hydrolysis of hemp biomass

The acid pretreated, washed, and dried hemp solids (1% w/v, dry basis glucan) were enzymatically hydrolyzed in a 50 mL capped bottle with a 10 mL of 50 mM citrate buffer (pH 4.8) combined with a measured amount of enzyme preparation. Four sets of the test were carried out in an orbital shaking incubator at 50 °C for 72 h with different enzyme loadings at 5, 10, 25, and 50 mg enzyme protein per g glucan, respectively. The liquid fraction from each hydrolysate sample was separated and filter-sterilized using a 0.45 μm nylon syringe filter and kept at 4 °C for further analysis. All experiments were tested in duplicate.

### Scanning electron microscopy (SEM)

The structural and morphological changes in hemp feedstock before and after pretreatment were observed using a JCM-6000 benchtop SEM equipment (JICM 6000-OG-2, JCM, Peabody, MA, USA) described in the previous work.^26^ A small piece of each stem and branch samples were placed onto an aluminum stub with double-sided carbon tape and coated with a gold–palladium alloy (Au/Pd) using a Cressington sputter coater (Ted Pella Inc., Redding, CA, USA). All SEM images were taken at 15 kV with a magnification of 500×.

### Analytical methods

After pretreatment soluble sugars, acetates, acids, and other soluble molecules were measured using HPLC equipment as described by Meng *et al.* (2020).^50^ The HPLC system employed an ion-exchange column and pulsed amperometic detection (HPAEC-PAD) in the ICS-3000 system (Dionex Corp., USA). Filter paper unit (FPU) activity in each enzyme was determined using Whatman filter paper strips.^28^ Endo-glucanase and β-glucosidase activities were analyzed using a CellG5 assay reagent (K-CellG5-2V, Megazyme, Wicklow, Ireland), and *para*-nitrophenyl β-d-glucopyranoside (*p*-NPG) as a substrate, respectively.^51^ For endo-glucanase, one enzyme activity unit is defined, as the amount of enzyme in the presence of excess thermostable β-glucosidase, required to release 1 μmol of 4-nitrophenol from the assay reagent. Similarly, the β-glucosidase activity was quantified with respect to the *p*-NPG substrate under the specified protocols.^51^ The protein concentration in each enzyme and glucose content from hydrolyzed samples were identified by a Pierce BCA protein assay kit (Thermo Scientific, IL, USA) and a glucose GOPOD assay kit (K-GLUC, Megazyme, Wicklow, Ireland). The profile of each enzyme activity and protein concentration are summarized in Table 3.

**Table:** (A) Profile of enzyme activity, and (B) generlized enzyme activities (unit per g substrate solids) at 10 mg protein per g substrate solids

Activity (units per mL)	Cellic Ctec2 (cellulase blend)	Multipect pectinase	Cellulase from *T. reesei*
(A)			
FPU^a^	50.4	4.2	3.5
Endo-G^b^	2179.9	326	156.7
β-G^c^	351.6	176	75.4
Protein (mg mL^−1^)	186.5	82.3	99.0

(B)			
FPU^a^	2.7	0.5	0.4
Endo-G^b^	116.9	39.6	15.8
β-G^c^	18.9	21.4	7.6

a Filter paper units (FPU mL^−1^).

b Endo-glucanase is defined as the amount of enzyme that required to release 1 μmol of 4-nitrophenol with assay reagent.

c β-gucosidase with respect to *p*-NPG. Generlized enzyme activity (unit per g substrate solids) = [enzyme activity per mg protein] × [enzyme loading (mg protein) per g substrate solids]^28^

## Results and discussion

### Impact of acid pretreatment on the chemical composition of hemp

In order to interpret the change of cellulose/hemicellulose/lignin components during the pretreatment, the chemical composition of pretreated solids was analyzed and summarized in Table 2A. Acid pretreatment was effective in increasing the glucan component both in hemp stems (from 39.6% to 52.6%) and branches (from 26.0% to 34.1%) by solubilizing a portion of hemicellulose (xylan) (Table 2A). Higher solubilization of the xylan fraction resulted in increasing glucan composition in the pretreated stem than those from the branch sample (52.5% *vs.* 4.2% xylan removal) while the total lignin contents were increased by 11.6% (from 26.0% to 29.4%) and 14.0% (from 30.6% to 35.6%) for stem and branch solids, respectively. It should be noted that a higher extent of lignin in the pretreated solids would be reflected by plant species, pretreatment type (*e.g.*, hydrothermal, dilute acid, ammonia fiber expansion), and severity factor (temperature and time). For instance, Ko *et al.* (2015) reported the higher lignin melting in hardwood achieved during LHW pretreatment between 170–180 °C.^52^ However, simply increasing temperature was not a suitable option because re-deposition of the lignin content on the hardwood surface was observed above 180 °C. Similar work done by Trevorah *et al.* (2018) elucidated that further lignin solubilization in *Eucalyptus via* γ-Valerolactone (GVL) pretreatment was available by increasing the temperature from 120 to 150 °C but less lignin removal was obtained at 180 °C.^53^ This is consistent with the current work for pretreatment at 121 °C for 30 min in order to minimize the generation of undesirable molecules and to avoid the re-aggregation of lignin onto the biomass. Monomeric sugars of glucose and xylose were identified in the pretreatment liquid fraction both in stems and branches while HMF and furfural were not formed (Table 2B). Acid pretreatment prompted the release of phenolic acids from stems (323.8 mg L^−1^) and branches (387.5 mg L^−1^), these were originated from some ester groups linked to hemicellulose and lignin degradation.

### Morphological changes of pretreated hemp solids

**Fig. 1:**
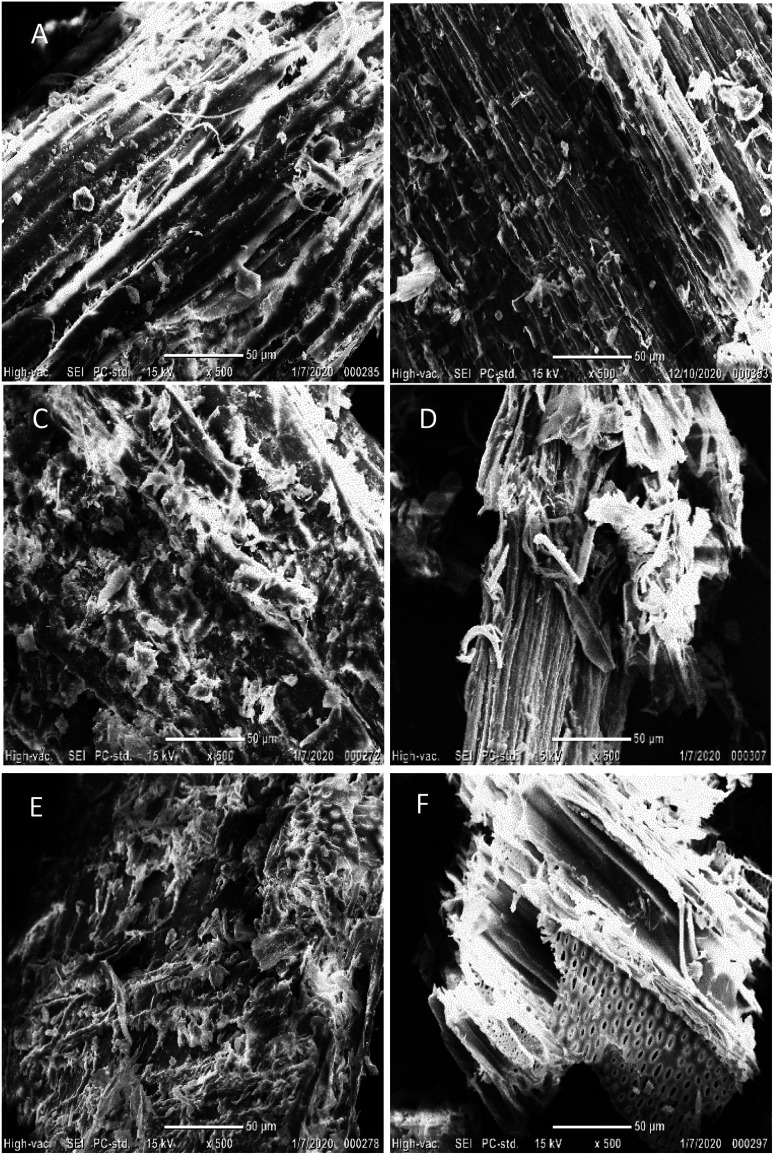
Scanning Electron Micrograph (SEM) images of untreated hemp materials, acid pretreated solids, and those hydrolyzed solids. (A) Raw hemp stems and (B) branches; (C) acid pretreated stem and (D) branch solids at 15% (w/v) solid in the presence of 1% (v/v) hydrochloric acid at 121 °C for 30 min; (E) enzymatically hydrolyzed acid-pretreated and washed stem and (F) branch solids. The images were taken at magnification of 500×. The scale bars and magnification information are presented at the bottom of each image.

The microstructural and morphological changes in hemp solids before/after acid pretreatment were captured through scanning electron micrograpy (SEM). The multiple layers of epidermis, xylem, phloem, and pith are organized in hemp stems, and branches are mainly composed of fibers.^54^ The flat and smooth surfaces of hemp structures were observed in the untreated hemp samples (Fig. 1A and B) but those pretreated solids were damaged and disrupted with erratic fragments (Fig. 1C and D). Pretreatment contributes toward the decreases in particle size, degree of polymerization (DP), and cellulose crystallinity while internal surface area and enzyme accessibility to cellulose are expanded. Representative physical changes of pretreated solids showed the disruption of external lignin coating and the exposure of internal contents to the outer cell wall areas (Fig. 1C and D). The revealed complex internal components are susceptible to being solubilized (mainly hemicellulose) and/or degraded into small molecules, but the remaining cellulose content exhibits a rough surface which allows a higher enzyme efficiency and conversion yield, providing enzyme adhesive sites for cellulose hydrolysis.^52,55,56^ Another noticeable change in the hydrolyzed hemp samples was the observation of rounded pith layers (small pores with a diameter of 1–5 μm) on the solids' surface (Fig. 1E and F). When the cellulolytic enzymes are mixed, the chemical linkages in the internal cellulose are broken down and degraded into monosaccharides, which can lead to the modification of hemp with the fibrillary matrix and vascular bundles.^26^

### Impact of cellulolytic enzymes on hemp hydrolysis

**Fig. 2:**
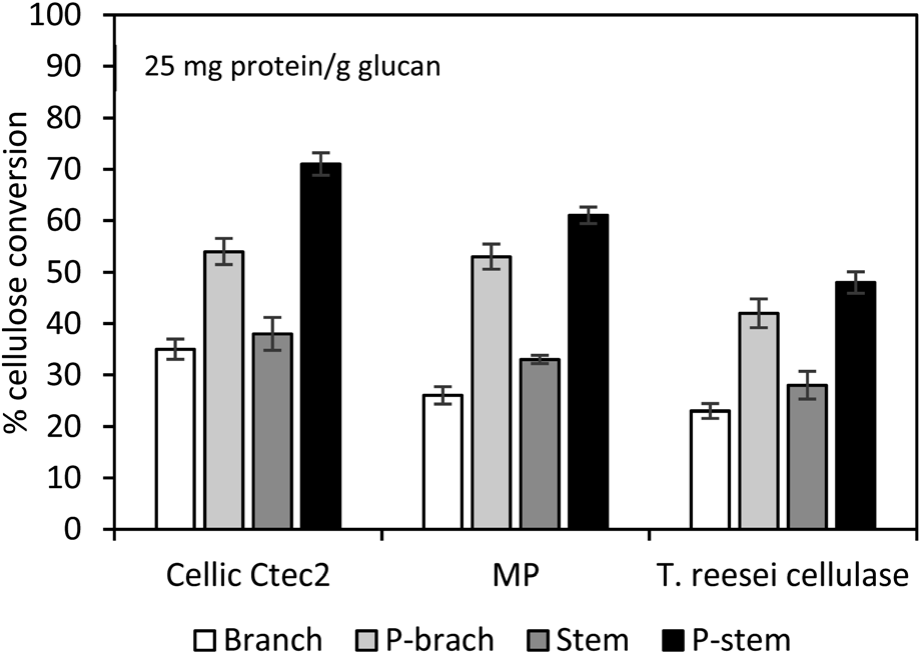
Cellulose conversion of washed pretreated hemp solids with different enzyme preparations at 25 mg protein per g glucan. Hydrolysis of 1% (w/v) glucan was conducted at 50 °C for 72 h with the agitation of 200 rpm. MP: Multipect Pectinase; P-stem: pretreated stem; P-branch: pretreated branch. The average conversion results in duplicate are presented and error bars indicate a confidence difference of 95%.

To verify the effect of cellulolytic enzymes on hemp hydrolysis, three different types of enzymes (Cellic Ctec2, Multipect Pectinase originating from *Aspergillus niger*, and cellulase from *T. reesei*) were evaluated and compared. Pretreated and washed hemp solids of 1 g dry glucan/100 mL solution were hydrolyzed using each enzyme preparation (25 mg enzyme protein per g glucan) at 50 °C for 72 h on an orbital shaker. Hydrolysis yields of control run with untreated stem and branch solids ranged from 23 to 38% glucan conversion, while the highest conversion yields were 71% (7.81 g L^−1^) and 54% (5.94 g L^−1^) for the pretreated stem and branch solids with Cellic Ctec2 (Fig. 2). Tests with Multipect Pectinase (MP) and *T. reesei* cellulase under similar experimental conditions generated 52% and 40% for stem solids, and 43% and 37% for the branch solids, respectively (Fig. 2). Using all three different enzyme tests, for both untreated and pretreated hemp samples, stem samples had higher yields compared to branch samples. Cellic Ctec2 was most effective for the cellulose conversion to glucose than those from the other two enzyme preparations. It is possible with significant differences in enzyme activities (Table 3). Higher activity values of endo-glucanase (Endo-G, 2179.9 units per mL) and β-glucosidase (β-G, 351.6 units per mL) in Cellic Ctec2 improved hydrolysis efficiency, compared to *T. reesei* cellulase (Endo-G: 156.7 and β-G: 75.4 units per mL) and MP (Endo-G: 326 and β-G: 176 units per mL). Previous literature also indicated that thermal denaturation was related to enzyme stability.^57,58^ The fact that β-G in Cellic Ctec2 was more resistant at 50 °C than those originating from *A. niger* may help explain the loss of enzyme activity.^59^

The overall results indicate that acid pretreatment was capable of structural/morphological changes in hemp materials and that subsequent hydrolysis with Cellic Ctec2 enzyme formulation was sufficient under the stated experimental conditions, to provide 1.4–1.9 times higher yields than raw samples. Besides the enzyme activities, non-productive bindings with undesirable molecules (*e.g.*, lignin-derived by-products) can cause enzyme inhibition.^44^ Solubilization and degradation of hemp biomass during pretreatment facilitates the formation of inhibitors/deactivators, mainly phenols in this work, which would impede the catalytic action of enzymes for cellulose hydrolysis. In order to alleviate the inhibitory effects on cellulose decomposition, the pretreated hemp solids were thoroughly washed with distilled water. However, insoluble molecules such as small lignin residues and lignin-derived molecules remained and may impact biomass recalcitrance. To better understand the non-productive binding of enzymes onto the lignin, further tests of the pretreated hemp solids were evaluated with bovine serum albumin (BSA), which plays a role as a lignin blocking agent, competes with lignin molecules, and allows more enzyme accessibility to cellulose.

### Cellulose hydrolysis in hemp biomass at different loadings

**Fig. 3:**
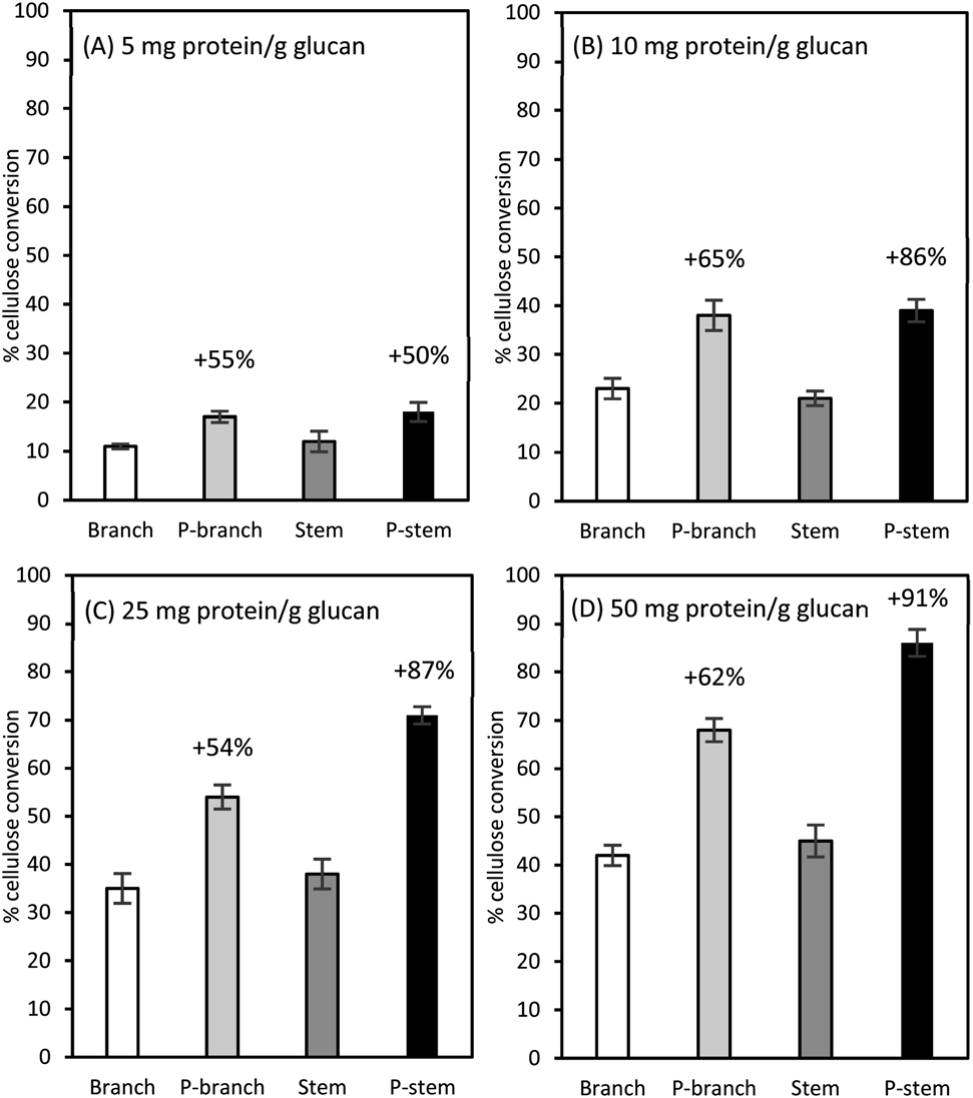
Percent cellulose conversion to glucose for unpretreated and pretreated hemp materials at a different enzyme dosage of Cellic CTec2 cellulase: (A) 5 mg protein per g glucan, (B) 10 mg protein per g glucan, (C) 25 mg protein per g glucan, and (D) 50 mg protein per g glucan. Cellulose in 1% (w/v) glucan solids was hydrolyzed in a 50 mL agitated flask at 50 °C for 72 h. All tests were run in duplicate and the average is presented. P-stem: pretreated stem; P-branch: pretreated branch.

Among the three enzymes employed, Cellic Ctec2 was selected for further assays at different enzyme loadings of 5, 10, 25, and 50 mg protein per g glucan (Fig. 3). As expected, the lowest glucose formations (less than 13%) were observed in the raw samples at 5 mg enzyme protein per g glucan while there was a 50% (from 12 to 18% in the stems) and a 54.5% (from 11% to 17% in branches) higher conversion yields in those pretreated samples (Fig. 3A). In the case of 10 mg protein per g glucan dosage for the pretreated solids, glucose recovery was increased from 21% to 39% for stems and from 23% to 38% for branch solids, which were comparable to the unpretreated samples at 25 mg protein per g glucan enzyme loadings (Fig. 3B and C). Likewise, at the highest enzyme loading of 50 mg g^−1^ glucan, the highest yields were observed in the pretreated samples (68% in branches, 86% in stems); however, the enzyme efficiency was superior to stem than branch samples (Fig. 3D).

These cellulose conversion results indicate that lignin content differences in biomass affected cellulose hydrolysis. The low lignin in pretreated and washed stem solids was more prone to be enzymatically degraded with less persistence (29.4% lignin in stems *vs.* 35.6% lignin in the branches, Table 3). This is consistent with the earlier work with switchgrass (*Panicum virgatum* L.) that down-regulation of a caffeic acid *O*-methyltransferase (COMT) gene in switchgrass enhanced the sugar recovery up to 34% and fermentation yield by up to 28% when compared with the control.^60^ They also elucidated that there was a remarkable decrease in S-lignin in the COMT down-regulated switchgrass, which consequently resulted in the lower total lignin content as well as an S/G lignin ratio. A recent study from our laboratory also demonstrated that, although there was a small difference in lignin composition in two different field-grown sugarcane bagasse clones (22.3% *vs.* 20.6%), the low lignin sample not only contributed less release of soluble inhibitors (mainly phenolic acids) during liquid hot water pretreatment but also yielded a higher saccharification efficiency than the high lignin pretreated bagasse solids with a 40 FPU g^−1^ glucan enzyme loading (68.3% *vs.* 89.5%).^26^ In this sense, the plant cell wall recalcitrance is significantly decreased by manipulating lignin biosynthesis and/or changing lignin content and ratio in the targeted crops.

### Effect of BSA for enzymatic hydrolysis

Pretreatment type and severity factor affect cellulose-hemicellulose-lignin composition in lignocellulose and influence the generation and distribution of inhibitors. Increasing the degree of the pretreatment severity factor not only affects the disruption of chemical linkages between cellulose-hemicellulose and cellulose-lignin but also results in re-deposition/re-aggregation of solubilized lignin on the surface of biomass and an increase in lignin-degraded small molecules.^42^ It is well-known that the molecules generated from the pretreatment are decisive inhibitors; in particular, lignin-derived aromatic alcohols (phenols) can significantly decrease the action of the enzymes by impeding their accessibility to the targeted cellulose. Qin and colleagues (2016) reported the inhibitory effect of vanillin, a major lignin-degraded phenolic molecule, on Avicel (lignin-free pure cellulose) hydrolysis such that the glucose conversion was decreased from 53% to 26% when the Avicel hydrolysis was tested in the presence of vanillin at 10 mg mL^−1^.^61^ They also addressed that increasing enzyme loading could partially alleviate the enzyme inhibition and enhance the conversion yield; however, raising inhibitor concentration was more severe to determine the cellulose conversion and inhibition degree. Another study of phenolic acids elucidated that the hydrolysis of 1% (w/v) Solka Floc (pure cellulose and hemicellulose) at 25 mg cellulase protein per g glucan in the presence of either citrate buffer or maple liquid hot water (LHW) pretreatment liquid fraction (rich in phenols) gave 92% and 40% glucose conversion, respectively. When enzyme loading was decreased from 25 to 1 mg protein per g glucan, 77% and approximately 30% conversion rates were observed in buffer and pretreatment liquid, respectively. This confirms a considerable enzyme inhibition and a decreased glucose conversion.^62^

**Fig. 4:**
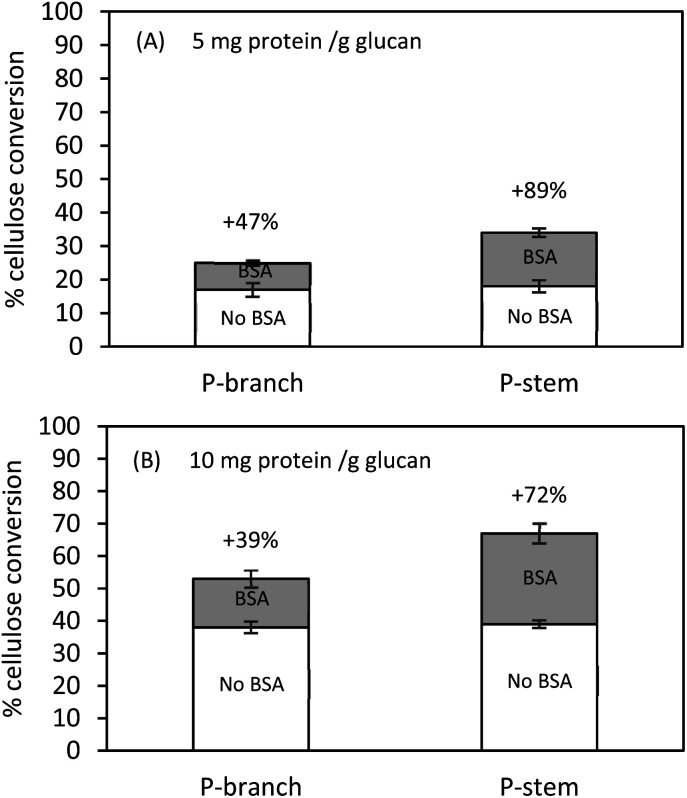
Cellulose conversion of pretreated and washed hemp solids at 1% (w/v) glucan in the presence of 5 or 10 mg protein per g glucan with or without supplementation. Hydrolysis was conducted in a 50 mL flask agitated (250 rpm) at 50 °C for 72 h. All tests were completed in duplicate with a 95% significant difference.

While pretreatment liquid was separated and potential soluble inhibitors in the solid fractions were washed away with distilled water, further investigations with BSA (bovine serum albumin, lignin blacking agent) were conducted to attempt to mitigate extensive inhibition by insoluble molecules (mainly lignin-derived compounds and lignin residuals), which are still present in the solids. Pretreated and washed hemp solids were pre-incubated in the presence of BSA (50 mg BSA g^−1^ solids) at 50 °C for 1 h. Thereafter, hydrolysis was performed with the cellulolytic enzyme at 5 mg and 10 mg protein per g glucan, respectively. It is worthwhile to mention that the higher enzyme dosage of 50 or 100 mg protein per g glucan could overcome the lignin-derived molecule inhibition and/or dilute the BSA effects on enzyme catalysis. Lower enzyme loadings (5 or 10 mg protein per g glucan) were applied for further tests. Lignin residues on the solid substrates were capable of adsorbing BSA supplemented to the liquid phase that reduced the non-specific binding of enzymes on lignin instead of cellulose. The overall conversion yields were increased in both BSA treated stem and branch solids compared to controls without BSA addition (Fig. 4).

Hydrolysis at 5 mg protein per g glucan after BSA treatment enhanced the conversion yield by 47% (from 17% to 25%) for branch and by 89% (from 18% to 34%) for stem solids (Fig. 4A). On the other hand, attempts using 10 mg protein per g glucan indicated that the addition of BSA improved the cellulose hydrolysis by 39% (from 38% to 53%) and 72% (from 39% to 62%) for branch and stem samples, respectively (Fig. 4B). These data suggest the alleviation effect of BSA on cellulose digestion, which was more effective on stem hydrolysis rather than branch solids, and it was relatively beneficial for a low enzyme loading instead of a high enzyme loading. Even though the higher conversion was achieved in the stem solids under 10 mg protein per g glucan, those tested at 5 mg protein per g glucan were more favorable (72% *vs.* 89% yield increase). The lignin composition after pretreatment may contribute toward the formation of lignin-derived residuals, the relatively lower lignin composition in the stem solids would release fewer inhibitors, for example, the total phenols shown in Table 2. The present results are in agreement with the previous studies which indicated the BSA would bind the exposed insoluble lignin-derived molecules and its subsequent hydrolysis enhancement is being preferred at low enzyme loading.^39,40,56^ The findings of Kim *et al.* (2017) for enzymatically fractionated corn pericarp, which has less than 5% lignin composition, gave 98% glucose conversion at 5 mg enzyme protein per g solids (equivalent to 22 mg protein per g cellulose) after 72 h.^28^ In comparison, in the case of sugarcane bagasse (>20% lignin) or mixed hardwood (>30% lignin), the enzyme inhibition and low conversion yield were observed, indicating the inhibitory effects by lignin content.^26,55,63^

It should be noted that the BSA supplementation is costly, and it could be substituted with a more cost-effective blocking agent such as protein isolated from the soybeans. In addition, the resulting enzyme inhibition and conversion yield in this study would be comprehensively ameliorated when they are performed with other operational considerations of biomass property, pretreatment type, severity factor, enzyme activity, additive concentration, pre-incubation time, and others.

## Conclusions

Hemp waste materials, which contain high cellulose and hemicellulose content, are potential alternatives for a sugar flatform process and renewable resources. Volatile compounds and soluble inhibitors from acid pretreated hemp could be removed by extensive washing with distilled water; however, the remaining insoluble lignin-derived inhibitors hamper the cellulolytic hydrolysis and decrease the sugar yield. Alleviation of these compounds in the solids by adding BSA before cellulose hydrolysis boosted the glucose yield both in stem and branch solids. The efficacy of BSA supplement suggests that stem solids containing lower lignin contents are more beneficial than those in branches at a lower enzyme loading, minimizing non-productive binding of enzyme proteins on inhibitors rather than exposed cellulose.

## Author contributions

Daehwan Kim: Conceptualization, Data curation, Formal analysis, Funding acquisition, Investigation, Writing – original draft, Writing – review & editing, Supervision. Chang Guen Yoo: Data Curation, Formal analysis, Writing – review & editing. Jurgen Schwarz: Funding acquisition, Writing – review & editing. Sadanand Dhekney: Funding acquisition, Writing – review & editing. Robert Kozak: Funding acquisition, Writing – review & editing. Craig Laufer: Writing – review & editing. Drew Ferrier: Formal analysis, Writing – review & editing. Skylar Mackay: Data curation, Formal analysis. Madyson Ashcraft: Data curation, Formal analysis. Richard Williams: Data curation, Formal analysis. Sinyeon Kim: Writing – review & editing.

## Funding

This research was supported by the Maryland E-Nnovation Initiative Fund (MEIF) funded by the Maryland Department of Commerce and the Maryland Energy Innovation Institute – 2020 Energy Innovation Seed Grant. The Summer Research Institute (SRI) funded by Hood College supported the student scholarships.

## Conflicts of interest

The authors declare that they have no known competing financial interests or personal relationships that could have appeared to influence the work reported in this paper.

## Supplementary Material
